# Effectiveness of Liraglutide in Patients With Obesity Who Are Early Non‐Responders to Lifestyle Intervention: A Real‐World Study

**DOI:** 10.1155/jobe/1224246

**Published:** 2026-08-27

**Authors:** Luisa Gilardini, Verdiana Vincenti, Marina Croci, Silvia Martinelli, Lucia Pasqualinotto, Paola Toja, Luca Cavaggioni, Simona Bertoli

**Affiliations:** ^1^ Obesity Unit – Laboratory of Nutrition and Obesity Research, Department of Endocrine and Metabolic Diseases, IRCCS Istituto Auxologico Italiano, Milan, Italy, auxologico.it; ^2^ Department of Theoretical and Applied Sciences, ECampus University, 22060, Novedrate, Italy, uniecampus.it; ^3^ Department of Food, Environmental and Nutritional Sciences (DeFENS), International Center for the Assessment of Nutritional Status (ICANS), University of Milan, Milan, Italy, unimi.it

**Keywords:** lifestyle intervention, liraglutide, obesity, weight loss

## Abstract

**Objective:**

This prospective, observational study examined the efficacy of liraglutide in subjects with obesity who did not experience early weight loss during a behavioral intervention in a real‐world setting.

**Methods:**

The weight loss intervention lasted for 12 weeks, and at Week 6, we offered liraglutide to subjects who had lost less than 2.5% of their body weight (early unresponder (eUR)). Before and after the intervention, anthropometry, body composition, blood pressure, level of physical activity, nutrition knowledge, quality of life, and daytime sleepiness were assessed.

**Results:**

Of 174 participants, 78 subjects were classified as early unresponders: 21 (27%) agreed to start liraglutide therapy (eUR‐LT), and 57 declined (eUR‐NLT), primarily due to the out‐of‐pocket cost. Liraglutide was well tolerated. Four subjects complained of nausea and two of epigastralgia. During the 6–12‐week period, the weight loss was −3.5 ± 1.8 kg (−3.2%) in the eUR‐LT group and −1.3 ± 1.6 kg (−1.4%) in the eUR‐NLT, *p* < 0.001. Using liraglutide therapy, baseline body mass index, sex, age, and weight change at 6 weeks as independent variables, the multivariate regression analysis found that only liraglutide therapy was significantly associated with weight loss from Week 6 to Week 12 (*p* < 0.001). After 12 weeks, total weight loss was −6.0 ± 2.4 kg (−5.3%) in group eUR‐LT, −2.4 ± 1.9 kg (−2.4%) in group eUR‐NLT, and −6.2 ± 2.3 kg (−6.3%) in group eR. In the eUR‐LT group, 66.7% lost ≥ 5% of the initial weight; in group eUR‐NLT, 14%; and in group eR, 72.9%.

**Conclusion:**

Liraglutide therapy is beneficial for individuals with obesity who do not respond early to lifestyle intervention. However, the high cost of the drug remains a barrier to its use.

## 1. Introduction

Obesity has reached epidemic proportions and has important consequences for morbidity, disability, and quality of life [1]. In Europe, individuals with overweight/obesity represent about 50% of the adult population [2]. A 5%–15% weight loss over a period of 6 months is realistic and has proven health benefits [3], but this goal is challenging to achieve. A lifestyle intervention is considered the first approach in obesity management. This should be multidisciplinary and includes diet, physical activity, cognitive behavioral therapy, and psychological support [4]. A 12‐week multidisciplinary intensive behavioral program, dedicated to individuals with obesity, has been conducted for many years at the Istituto Auxologico Italiano in Milan. At the conclusion of the intervention, we observed a mean weight loss of approximately 5% and a significant improvement in body composition, blood pressure [5], insulin resistance, minimal nocturnal SaO_2_ [6], and nutrition knowledge [7]. However, there is a large variability in response, with some individuals being highly successful while others lose very little weight. Moreover, it is known that larger initial weight loss is associated with larger long‐term weight losses [8]. The factors underlying early resistance to weight loss are not fully understood. Some studies have identified female gender, non‐Hispanic White ethnicity, poor baseline physical activity, eating‐disorder psychopathology, and depressive symptoms as predictors of low early weight loss [9, 10]. If the lifestyle intervention fails, the guidelines recommend considering pharmacotherapy, such as a glucagon‐like peptide‐1 (GLP‐1) receptor agonist, that induces weight loss by delaying gastric emptying and increasing satiety [11]. A recent meta‐analysis of 18 randomized controlled trials reported that, compared with placebo, liraglutide was associated with a 4.7% weight reduction among adults with obesity [12].

In this real‐world study, we developed a strategy to achieve a 5% weight loss goal, identifying individuals who did not respond early to the lifestyle program (< 2.5% weight loss after 6 weeks) and offering them liraglutide therapy, the sole GLP‐1 receptor agonist available in Italy for the treatment of obesity at that time. The threshold was intended as an early predictive marker for subsequent treatment success rather than a definitive therapeutic endpoint and was chosen at 2.5% because it represents half of the 5% weight loss that is associated with improved health outcomes by the end of treatment. In addition, several studies have previously used a 2%–2.5% threshold to identify poor weight loss response [9, 10, 13].

## 2. Methods

This study included consecutive patients with obesity who were referred to the IRCCS Istituto Auxologico Italiano in Milan, a specialized center for the study of obesity in adults and children, for a 12‐week multidisciplinary lifestyle intervention program from September 2023 to April 2024. Inclusion criteria were age between 18 and 70 years and body mass index (BMI) ≥ 30 kg/m^2^. Exclusion criteria were secondary obesity, uncompensated hypothyroidism, cognitive impairment, and inability to engage in physical activity. Before and 12 weeks after the intervention, weight, height, BMI, waist circumference, body composition, blood pressure, physical activity levels, nutrition knowledge, quality of life, and daytime sleepiness were assessed.

At Week 6 of rehabilitation, we evaluated in all subjects the weight loss obtained. Patients who had not achieved a weight loss greater than 2.5% (we defined them as early unresponders, eUR) were offered liraglutide therapy. Liraglutide was self‐administered once daily by subcutaneous injection. The initial dose was 0.6 mg. To minimize gastrointestinal adverse events, the dose was escalated to 1.2 mg at week 7 and to 1.8 mg at week 9 and maintained at 1.8 mg until Week 12. Liraglutide was introduced after Week 6 and continued until Week 12, resulting in a pharmacological exposure of only six weeks. Every week, the doctor monitored adherence to the therapy and the occurrence of side effects. Patients received specific counseling on managing potential side effects to improve medication adherence. Subjects who started liraglutide were defined as early unresponders‐liraglutide therapy (eUR‐LT), and those who did not accept liraglutide therapy were defined as early unresponders‐no liraglutide therapy (eUR‐NLT). Subjects who obtained ≥ 2.5% weight loss with lifestyle intervention at Week 6 were defined as early responders (eR).

Participants were weighed to a precision of 0.1 kg on a calibrated mechanical beam scale, wearing minimal indoor clothing and barefoot. Height was recorded to the nearest 0.5 cm using a wall‐mounted stadiometer. BMI calculation followed the standard formula of weight divided by height squared. An inelastic measuring tape was employed to assess waist circumference at the midpoint between the last rib and the iliac crest, to the nearest 0.5 cm. To determine body composition, bioelectrical impedance analysis was performed using an Akern BIA 101‐RJL system (Akern srl, Firenze, Italy). The evaluation was performed in the morning after resting for 20 min in a temperature‐regulated, well‐ventilated setting. Participants were strictly instructed to maintain a minimum 5 h fast and to abstain from physical activity in the previous 12 h. Blood pressure measurement involved a series of three separate readings obtained at 5 min intervals while the participant was seated quietly. Assessments were taken with a standard mercury sphygmomanometer and a cuff size optimized for arm circumference. Systolic and diastolic values were determined using Korotkoff sounds phases I and V, respectively.

The Moynihan Questionnaire was used to evaluate dietary knowledge. Final scores of this tool range from 15 to 30, and a lower score corresponds to higher nutrition knowledge [14].

Daytime sleepiness was monitored through the Epworth Sleepiness Scale (ESS), which yields a score range from 0 to 24. A score equal to or exceeding 10 points indicates excessive daytime sleepiness [15].

Physical activity was quantified utilizing the short form of the International Physical Activity Questionnaire (IPAQ) that calculates the Metabolic Equivalent of Task (MET: time spent in physical activity, expressed in minutes per week) [16].

The overall self‐reported quality of life was measured using the EuroQol five‐dimensional visual analogue scale (EQ‐5D VAS). This instrument utilizes a 20‐cm vertical scale ranging from a score of 0 (representing the worst imaginable health state) to 100 (representing the best imaginable health state), upon which patients indicate their perceived current health status [17].

### 2.1. Lifestyle Intervention

The 3‐month lifestyle intervention consisted of weekly comprehensive center visits for nutritional education, behavioral therapy, and physical activity in the gym supervised by a certified sport scientist. Each session lasted about 4 hours and included individual evaluations and group sessions. Following a baseline medical and nutritional assessment, the dietitian designed an individualized hypocaloric balanced diet with 20% protein, 30% fat, and 50% carbohydrates. The diet provided a mean deficit of 800 kcal/day compared to the individual caloric requirement estimated by the Mifflin equation and corrected for the individual physical activity factor. Adherence to both the nutritional targets and physical training schedules was enforced and reinforced through a detailed self‐monitoring diary, in which participants recorded daily food consumption, physical activity, and contextual emotional triggers. During individual interviews, the dietician and the physician monitored the weight changes, discussed the self‐monitoring diary, reinforced beneficial dietary adjustments, and provided suggestions to avoid or limit food habits that may compromise the weight reduction. In addition, patients participated in nutritional group sessions based on an interactive method of teaching/learning and focused on various topics, such as healthy eating principles, the Mediterranean food pyramid, and strategies for managing meals out and social events. Specific educational blocks were dedicated to exploring the pathophysiological mechanism of obesity, its clinical sequelae, and available medical management options, with an explicit focus on the physiological pathways, potential contraindications, and adverse profile of anti‐obesity medications. The rehabilitation program included cognitive behavioral therapy carried out through group sessions and individual psychological consults when clinically indicated. During the sessions, the psychologist motivated the patients to change the incorrect behaviors of their lifestyle and provided several procedures, such as setting realistic and achievable goals, control of dangerous stimuli, self‐monitoring during eating, and adopting alternative behaviors during critical emotional situations or negative mood states. Finally, at each occasion, the patients attended a one‐hour session of moderate‐intensity aerobic physical activity integrated with muscle‐strengthening activity. Personalized home‐based physical activity guidelines were also prescribed, taking into account individual baseline functional capacities.

### 2.2. Ethical Statement

This study was conducted in accordance with the ethical standards stipulated in the Declaration of Helsinki and was approved by the Ethics Committee of the Istituto Auxologico Italiano on 22nd November 2022 (reference number: 2022_11_22_04). Written informed consent was obtained from all participants.

### 2.3. Statistical Analysis

All data are expressed as mean ± standard deviation (SD) for continuous variables or as percentages for categorical variables. The Shapiro–Wilk test was used to assess the normality of the data distribution. Analysis of variance was carried out to compare differences among groups in baseline values. The group frequencies were compared using a chi‐square test. A paired‐samples *t*‐test was used to compare variables before and after intervention. Multivariate linear regression analysis was used to assess the determinants of weight loss between Week 6 and Week 12 in group eUR‐LT and eUR‐NLT. Prior to executing the multivariate regression analysis, all underlying statistical assumptions were formally verified. Multicollinearity among predictors was assessed using the variance inflation factor (VIF); all obtained VIF values were below 2 (range: 1.033–1.456), indicating no significant collinearity. Independence of residuals was confirmed by a Durbin–Watson statistic of 1.964, and residual diagnostics demonstrated a proper distribution centered around a mean of 0.0, with standardized residuals safely within the −2.63 to 2.27 range. Finally, overall model fit adequacy was validated by the omnibus F‐test (*F* = 5.822, *p* < 0.001, *R* ^2^ = 0.254).

The significance level for all tests was set at *p* < 0.05. All analyses were performed using SPSS software, version 28.0 (IBM Corp., Armonk, NY, USA).

## 3. Results

**FIGURE 1:**
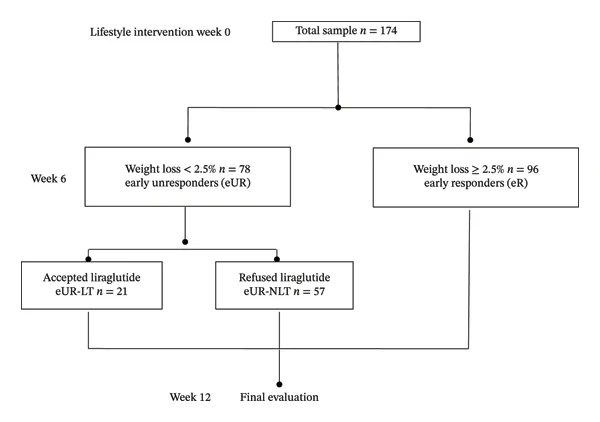
Participant treatment flow diagram. eR: subjects early responders (weight loss ≥ 2.5% at Week 6) to lifestyle intervention; eUR: early unresponders (weight loss < 2.5% at Week 6); eUR‐LT: subjects eUR who initiated liraglutide at Week 6; eUR‐NLT: subjects eUR who declined liraglutide therapy.

Between September 2023 and April 2024, 174 individuals with obesity participated in the 12‐week lifestyle intervention. All subjects completed treatment. None of the subjects recruited had obesity induced by endocrinologic disorders. Participants receiving treatment for hypothyroidism had their thyroid hormone replacement stable for at least 3 months and thyroid‐stimulating hormone (TSH) levels within the normal reference range. At Week 6, 96 subjects (55.2%) obtained a weight loss of ≥ 2.5%. Subjects who lost weight successfully (eR) had a lower baseline BMI than those who did not (eUR) (36.8 ± 5.8 vs 39.4 ± 5.8 kg/m^2^, *p* < 0.005). Age and sex were not significantly associated with weight loss. We proposed therapy with liraglutide to eUR subjects (*n* = 78), and 21 (27%) of them accepted Figure 1. The most common reasons for refusing pharmacological therapy are shown in Table 1.

**TABLE 1:** Reasons for declining liraglutide therapy among early nonresponders (*n* = 57).

Reasons for declining therapy	Percentage of subjects
Out‐of‐pocket cost	60%
Fear of side effects	20%
Perceiving obesity as a disease not requiring medical treatment	10%
Distrust of drug therapies	5%
Fear of subcutaneous injections	5%

Clinical characteristics of the three groups studied are shown in Table 2.

**TABLE 2:** Baseline clinical characteristics of 174 subjects attending a 12‐week lifestyle intervention.

	Group eUR‐LT (*n* = 21)	Group eUR‐NLT (*n* = 57)	Group eR (*n* = 96)
Age, years	52.8 ± 12.4	52.9 ± 14.6	56.0 ± 10.3
Sex (female), %	81.0	87.7	89.6
BMI, kg/m^2^	43.2 ± 7.4^∗∗^	37.9 ± 4.4	36.8 ± 5.8
Waist circumference, cm	125.5 ± 9.6^a^	118.2 ± 11.7	117.7 ± 11.3
Fat mass, %	51.5 ± 5.6	50.0 ± 5.4	48.8 ± 5.6
Systolic BP, mmHg	135.7 ± 13.6^∗^	126.2 ± 11.5	123.9 ± 15.8
Diastolic BP, mmHg	86.2 ± 11.2^∗^	80.5 ± 7.4	80.7 ± 8.3
Hypertension, %	66.7	52.6	49.0
Dyslipidemia, %	52.4	42.1	32.3
Diabetes, %	9.5	14.5	11.5
Obstructive sleep apnea, %	19.0	17.5	9.4
Cardiovascular disease, %	4.8	8.8	8.3
Psychiatric disorders, %	9.5	28.1	17.7
Orthopedic disease, %	28.6	14.0	9.4
Moynihan score	21.2 ± 3.0	21.7 ± 3.1	21.2 ± 3.7
ESS score	4.9 ± 3.5	5.3 ± 3.8	6.3 ± 4.3
METs	339.9 ± 87.1	500.7 ± 174.4	370.6 ± 37.6
EQ‐5D VAS	64.8 ± 16.6	60.7 ± 16.3	64.7 ± 16.6

*Note:* eUR‐LT: group did not achieve < 2.5% weight loss and began liraglutide therapy; eUR‐NLT: group did not achieve < 2.5% weight loss and declined liraglutide therapy; eR: group achieved ≥ 2.5% weight reduction with lifestyle intervention at Week 6.

Abbreviations: BMI, body mass index; BP, blood pressure; ESS, Epworth Sleepiness Score; METs: metabolic equivalent; EQ‐5D VAS: EuroQol five‐dimensional visual analogue scale.

^a^ *p* < 0.05 vs eR group.

^∗∗^ *p* < 0.001, ^∗^ *p* < 0.05 vs eUR‐NLT and eR group.

Subjects who started liraglutide (eUR‐LT) had significantly higher BMI and blood pressure than subjects who did not, and were of a similar age and proportion of females. Moreover, they had a higher prevalence of hypertension, dyslipidemia, orthopedic disease, and obstructive sleep apnea, although not statistically significant.

Liraglutide was well tolerated. Six subjects needed to remain at a dose of 1.2 mg for 3 weeks before escalating to the next dose due to mild adverse events. Four subjects complained of nausea and two of epigastralgia. At the end of the rehabilitation, all subjects in pharmacological treatment reached a liraglutide dose of 1.8 mg/day. Liraglutide was introduced after Week 6 and continued until Week 12, resulting in a pharmacological exposure of only six weeks.

**FIGURE 2:**
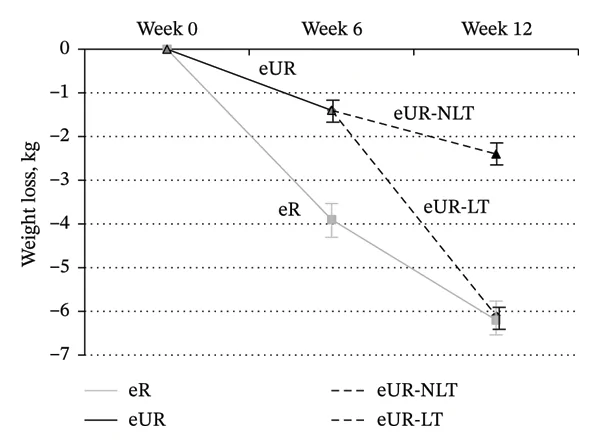
The gray line represents absolute weight loss at Week 6 and Week 12 in subjects early responders (eR, *n* = 96). The solid black line represents absolute weight loss at Week 6 in subjects who did not achieve 2.5% weight loss (early unresponders, eUR, *n* = 78). The dotted line and dashed line represent absolute weight loss at Week 12 in subjects eUR who initiated liraglutide (early unresponders–liraglutide therapy, eUR‐LT, *n* = 21) and who declined liraglutide (early unresponders–no liraglutide therapy, eUR‐NLT, *n* = 57), respectively. Values represent mean ± SE.

Figure 2 represents the absolute weight loss at Week 6 and Week 12 in the three groups.

After 12 weeks, weight loss was −6.0 ± 2.4 kg (−5.3%) in group eUR‐LT, −2.4 ± 1.9 kg (−2.4%) in group eUR‐NLT, and −6.2 ± 2.3 kg (−6.3%) in group eR. In the eUR‐LT group, 66.7% lost ≥ 5% of the initial weight; in group eUR‐NLT, 14%, and in group eR, 72.9%.

At 12 weeks, there was a decrease in waist circumference and fat mass in the three groups (Δwaist: −5.4 ± 2.8 cm in eUR‐LT, −2.8 ± 2.5 cm in eUR‐NLT, and −5.1 ± 3.2 cm in eR; Δ fat mass: −1.3 ± 3.4% in eUR‐LT, −0.7 ± 3.6% in eUR‐NLT, and −2.3 ± 5.1% in eR).

Since all subjects with obesity received the same treatment in the first six weeks, we considered the weight loss between Week 6 and Week 12 in group eUR‐LT and in group eUR‐NLT. The weight loss (6–12 weeks) was −3.5 ± 1.8 kg (−3.2%) in the eUR‐LT group and −1.3 ± 1.6 kg (−1.4%) in the eUR‐NLT group, *p* < 0.001.

Multivariate linear regression analysis was used to assess the determinants of weight loss between Week 6 and Week 12 in group eUR‐LT and eUR‐NLT (Table 3). Using Δweight at weeks 6–12 (weight at Week 12—weight at Week 6) as the dependent variable and liraglutide therapy, baseline BMI, sex, age, and weight loss at Week 6 as independent variables, only liraglutide therapy remained significantly correlated with Δweight at Weeks 6–12 (*p* < 0.001).

**TABLE 3:** Multivariate linear regression analysis of determinants for weight change between Weeks 6 and 12.

Independent variables	Coefficient (B)	SE	Standardized coefficient (β)	*p*	t value	CI 95%
Liraglutide therapy	−1.845	0.510	−0.434	< 0.001	−3.616	−2.967; −1.317
Δ weight at Week 6	0.019	0.155	0.014	0.903	0.122	−0.290; 0.328
Age	−0.005	0.040	−0.035	0.736	−0.339	−0.333; 0.023
Baseline BMI	−0.059	0.036	−0.182	0.107	−1.634	−0.131; 0.013
Sex	0.596	0.548	0.110	0.281	1.087	−0.497; 1.688

*Note:* Dependent variable: Δweight (weight at Week 12‐weight at Week 6). Model summary: *R* 0.504, *R* ^2^ 0.254, Adjusted *R* ^2^ 0.244, Model *F* = 5.822, *p* < 0.001. VIF index < 2 for all variables. Durbin–Watson statistic = 1.964.

Abbreviations: CI, confidence interval; SE, standard error; VIF, variance inflation factor.

At Week 12, there was a significant improvement in physical activity (METs: 339.9 ± 87.1 vs 857.4 ± 92.1 in eUR‐LT group and 500.7 ± 174.4 vs 973.8 ± 178.1 in eUR‐NLT, *p* < 0.001 for both) and food knowledge (Moynihan score: 21.2 ± 3.0 vs 19.3 ± 3.2 in group eUR‐LT and 21.7 ± 3.1 vs19.8 ± 2.9 in group eUR‐NLT, *p* < 0.001 for both), both in groups eUR‐LT and eUR‐NLT.

## 4. Discussion

Multiple clinical trials have shown that the use of liraglutide compared with placebo leads to greater total body weight loss and to a greater proportion of patients achieving at least 5% weight loss [12, 18, 19]. Real‐world studies provide the opportunity to evaluate the efficacy of the pharmacological therapy in routine clinical practice, where clinical complexities arise. For instance, about 20% of the subjects recruited in our study had psychiatric disorders, a condition that is often an exclusion criterion in clinical trials, but that is highly prevalent in patients with obesity.

In this study, we chose to depart from standard clinical practice by evaluating an “early triage strategy tailored for lifestyle non‐responders.” Specifically, we proposed initiating liraglutide therapy in individuals who had lost less than 2.5% of their initial weight very early, after only 6 weeks of lifestyle intervention. In fact, evidence from the literature indicates that early weight‐loss trajectories during lifestyle interventions are among the strongest predictors of long‐term treatment success in obesity management, with early non‐response identifying individuals at a higher risk of subsequent treatment failure [8, 20]. This threshold was intended as an early predictor of subsequent treatment response rather than as a definitive therapeutic endpoint. We also decided to initiate pharmacological therapy after 6 weeks, rather than the 12 weeks recommended by current guidelines, in order to allow for closer patient follow‐up through weekly visits and more careful monitoring of adverse effects. In addition, we established slowly titrating the dose of liraglutide to reduce gastrointestinal side effects and reach a dose of 1.8 mg at Week 12.

After 6 weeks of intervention, 45% of participants lost less than 2.5% of their weight and were therefore classified as early non‐responders to lifestyle modification. Although liraglutide was offered to all these patients, approximately 73% declined pharmacological treatment. There is limited literature on this issue, with varying results. A recent study estimated the rate of primary no adherence to anti‐obesity medications among patients who are newly prescribed and found that 84.5% of patients did not fill their new prescription for liraglutide [21]. In a French real‐life study, the percentage of subjects who never initiated the prescribed therapy with liraglutide was 25% [22]. In this study, as well as in ours, the major reason for not starting liraglutide included cost, followed by concerns about potential side effects.

The cost of the drug represents a major barrier to treatment access; in fact, liraglutide was not covered by the Italian National Health Service, and one month of liraglutide at a 3.0 mg dose cost about 250 EUR, an amount that is unaffordable for many individuals in Italy. Considering that in high‐income countries, lower socioeconomic position is associated with higher BMI [23], it is evident that only a few people can currently benefit from liraglutide. Globally, pharmaceutical pricing and disparities in financial resources hinder the equitable distribution of pharmaceutical innovations like anti‐obesity medications. Two large cross‐sectional analyses have shown that among U.S. adults, Black adults, Hispanic adults, and adults of lower socioeconomic status had a lower receipt of GLP‐1 agonist for treatment of obesity [24, 25]. The future availability of generic liraglutide could improve these existing inequities in obesity treatment. Socioeconomic status may have played a role in patients′ decisions regarding the initiation of pharmacological therapy; however, data on this variable were not available, preventing us from assessing its potential impact.

The subjects who initiated liraglutide had a higher degree of obesity and higher prevalence of comorbidities than those who declined pharmacological therapy. It is plausible that a worse medical condition has motivated these subjects to take anti‐obesity medication in addition to lifestyle changes. Strong evidence suggests that GLP‐1RA treatment had positive effects on obstructive sleep apnea syndrome outcomes [26] and hypertension [19]. There is only one study investigating the effect of liraglutide on knee pain. In this study, liraglutide was randomly added after 8 weeks of dietary intervention and a mean weight loss of 12 kg. The knee pain improved significantly during the run‐in phase of the study, but at the end of the study, there was no difference in further improvement between the liraglutide and placebo group [27]. Although participants with more severe obesity appeared to be more motivated to lose weight, they did not achieve clinically meaningful weight loss with lifestyle intervention alone and showed significant improvement after the initiation of liraglutide. This observation suggests that pharmacological treatment was a major contributor to the subsequent weight loss. Nevertheless, it is also possible that the introduction of liraglutide increased patients′ motivation and engagement with lifestyle recommendations, further enhancing treatment outcomes.

At the end of the 12‐week lifestyle intervention, subjects receiving liraglutide therapy had significant mean weight loss of −6.0 kg (−5.3%), greater than those who had not started pharmacotherapy. Moreover, 66.7% of the eUR‐LT group lost ≥ 5% of the initial weight. A direct comparison between our findings and those of clinical trials is not possible, since trial populations and designs differed. In any case, after 6 weeks of therapy, weight loss was about 4% with liraglutide, 3% with semaglutide, and 5% with tirzepatide in clinical trials [18, 28, 29]. It is difficult to compare our results with other real‐life studies, because the patients had been on therapy for only 6 weeks and the maximum dose reached was 1.8 mg. In a Swiss cohort, 42% of subjects reduced their weight by ≥ 5% and had a weight loss of −4.4 kg after 4 months with a median dose of liraglutide of only 1.5 mg/d [30]. Compared with our study, average weight loss was slightly lower in this study, probably because our subjects participated in an intensive and multidisciplinary treatment, in addition to drug therapy. A retrospective, observational Spanish study evaluated the effectiveness of liraglutide in patients with overweight or obesity and insufficient weight loss after a 6‐month lifestyle modification program [31]. After 3–6 months of treatment, mean weight loss was −6.4 kg and 57% of individuals lost at least 5% of their baseline weight.

In our study, the main determinant of weight loss between 6 and 12 weeks in eUR‐LT and eUR‐NLT groups was drug therapy. At the end of the treatment, the level of physical activity and the improvement of food knowledge were similar between the groups. We didn’t collect data on diet compliance in the two groups, but the effect of liraglutide is certainly mediated by reduced appetite and energy intake [5]. Indeed, liraglutide induces postprandial satiety and fullness, slows gastric emptying, and decreases appetite and food consumption by acting on the hypothalamus, limbic/reward system, and cortex [32].

The main limitation of the present study is that it was conducted at a single center, resulting in a small sample size that limits the statistical power, the precision of estimates, and the generalizability of the findings. Although the program was covered by the national health system, it is possible that our cohort is not also representative from a socioeconomic and cultural perspective. In fact, Milan is the Italian city with the highest gross domestic product and the highest education level. Although this has allowed for the standardization of patient management practice, further studies are needed, involving larger populations and multi‐center studies.

Another limitation may be the short duration of the treatment and the lack of long‐term follow‐up data. At the end of the program, subjects were given an appointment for regular 3‐month follow‐up visits. A future objective of our research is to collect these follow‐up data to explore whether the benefits of liraglutide are maintained in the long term. It would also be of interest to investigate whether the subjects taking anti‐obesity drugs have greater adherence to follow‐up visits than those managed with lifestyle intervention alone. This could be another advantage in intervening early with drug therapy in the treatment of obesity, a chronic disease that is characterized by high rates of treatment discontinuation.

Moreover, we have prescribed only liraglutide because when the study began, there were no other GLP1 receptor agonists on the market. In the meantime, semaglutide and tirzepatide have become available. We will continue the study by offering patients these new drugs.

## 5. Conclusion

Our real‐world study showed that individuals with severe or morbid obesity who did not respond early to lifestyle intervention benefited from liraglutide therapy. These findings support the concept that obesity treatment should be tailored and pharmacological therapy should be considered early when lifestyle intervention alone does not achieve the expected weight‐loss targets.

The out‐of‐ pocket cost of liraglutide remains a barrier to its use. Our findings therefore highlight the need for policy measures aimed at improving access to effective pharmacological treatments for obesity and reducing inequalities in obesity care.

## Funding

This study was funded by the Ministry of Health.

## Disclosure

The funders had no role in the study design, data collection and analysis, decision to publish, or preparation of the manuscript.

## Conflicts of Interest

The authors declare no conflicts of interest.

## Data Availability

The dataset used during the current study is available from the corresponding author on reasonable request, subject to compliance with ethical and privacy regulations.
